# 76. Effects of the “Undetectable = Untransmittable” (“U=U”) Educational Campaign on Treatment Outcomes and Perceptions among People Living with HIV in North American Countries

**DOI:** 10.1093/ofid/ofab466.076

**Published:** 2021-12-04

**Authors:** Frank Spinelli, Bruce Richman, Patricia De Los Rios, Benjamin Young, Marvelous Muchenje, Nicolas Van de Velde, Chinyere Okoli

**Affiliations:** 1 ViiV Healthcare, Sag Harbor, NY; 2 Prevention Access Campaign, New York, New York

## Abstract

**Background:**

The educational campaign “Undetectable = Untransmittable” (U=U) began in 2016 to improve the well-being of people living with HIV (PLHIV) and recalibrate HIV-related social norms. As medical practice can vary by region, we examined reports from PLHIV in North American countries to identify if the campaign affected healthcare provider (HCP) communication of U=U and if positive health outcomes differed by U=U-informed status or country.

**Methods:**

Data were collected from the 2019 Positive Perspectives survey of PLHIV in Canada (n=120), Mexico (n=63), and the United States (US; n=400) and stratified by country. Outcomes were self-rated mental and sexual health (“Good”/”Very good”), viral suppression, and sharing of HIV status. Treatment perceptions were also assessed.

**Results:**

Whether diagnosis occurred before or after the U=U campaign launch did not significantly affect the proportion of PLHIV who reported receipt of U=U from an HCP in any North American country. Whether an individual was informed of U=U varied significantly by sexual orientation (heterosexual, 62.8%; homosexual, 74.9%; other, 87.8%), sex (men, 64.7%; women, 89.8%; other, 100%), and metropolitan vs non-metropolitan residence (metropolitan, 78.2%; non-metropolitan, 65.2%) in the US (*P*< 0.01 for all) but not in Canada or Mexico. Prevalence of sharing of HIV status with ≥ 1 person besides an HCP varied among PLHIV (Canada, 87%; Mexico, 95%; US, 84%). Prevalence of other positive outcomes varied by country and whether PLHIV had been informed of U=U (Figure). Whether PLHIV were informed of U=U was also correlated with treatment satisfaction and the perception that treatment needs were being met among PLHIV in the US and Canada (*P*< 0.01 for all), and with the belief that treatment prevents transmission among PLHIV in the US (*P*=0.01).

Figure. Prevalence of positive outcomes among PLHIV in Canada (n=120), Mexico (n=63), and the US (n=400). PLHIV, people living with HIV; US, United States; U=U, Undetectable = Untransmittable. *P<0.01 for U=U informed vs uninformed.

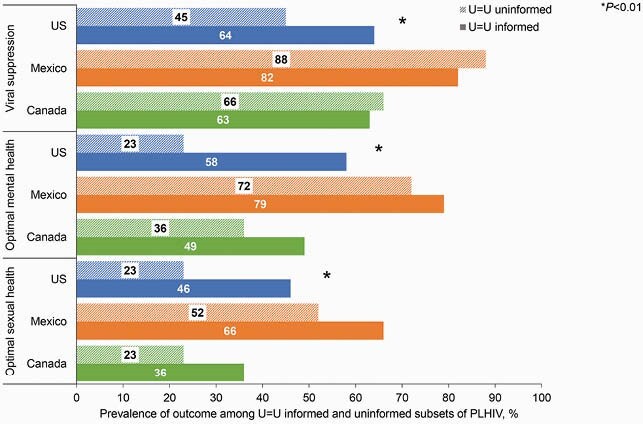

**Conclusion:**

Being informed of U=U was associated with higher treatment satisfaction, and higher mental and sexual health outcomes among PLHIV in North America. Receipt of U=U was associated with significantly higher treatment satisfaction among PLHIV in the US and Canada, and significantly more PLHIV with optimal mental and sexual health in the US.

**Disclosures:**

**Frank Spinelli, MD**, **ViiV Healthcare** (Employee) **Bruce Richman, JD**, **ViiV Healthcare** (Consultant) **Patricia De Los Rios, MSc**, **GlaxoSmithKline** (Shareholder)**ViiV Healthcare** (Employee) **Benjamin Young, MD, PhD**, **GlaxoSmithKline** (Shareholder)**ViiV Healthcare** (Employee) **Nicolas Van de Velde, PhD**, **ViiV Healthcare** (Employee) **Chinyere Okoli, PharmD, MSc, DIP**, **GlaxoSmithKline** (Shareholder)**ViiV Healthcare** (Employee)

